# Online Advanced Analytical Service: Profiles for Dengue Hemorrhagic Fever Transmission in Southern Thailand

**Published:** 2019-11

**Authors:** Siriwan KAJORNKASIRAT, Jirapond MUANGPRATHUB, Nathaphon BOONNAM

**Affiliations:** Faculty of Science and Industrial Technology, Prince of Songkla University, Surat Thani Campus, Surat Thani, Thailand

**Keywords:** Dengue Hemorrhagic Fever (DHF), Spatio-temporal, Advanced analytic, Google Maps™

## Abstract

**Background:**

Southern Thailand has the highest Dengue Hemorrhagic Fever (DHF) incidence and fatality rate in Thailand. Geographic Information Systems (GIS) technology and spatial analysis techniques are powerful tools to describe epidemiological patterns. The aim of this study was to develop an Online Advanced Analytical Service: Profiles for Dengue Hemorrhagic Fever Transmission (OSD) in Southern Thailand.

**Methods:**

The system was developed using JavaServer Pages (JSP) and Database Management System (DBMS) with Structured Query Language (SQL) technology as the web database tool for data entry and data access, web *Mathematica* technology for data analysis and Google Maps™ API technology for online data display as the map service implementing GIS technology.

**Results:**

The OSD system has been available online at URL http://www.s-cm.co/dengue . Users performed data entry using the web-service with login by social network (i.e. Facebook) account, used data analysis tools with online real-time statistical analysis and data display with transparent color circles overlaid on Google Maps™.

**Conclusion:**

The OSD system display represents the distribution of DHF cases with spatial information. This system enables health planners to provide interventions for DHF focusing on prevention, control, and strategic planning.

## Introduction

Dengue Hemorrhagic Fever (DHF) is a vector borne disease transmitted to human through the bites of infective female *Aedes* sp. (Diptera: Culicidae) mosquitoes (1, 2), principally by *Ae. aegypti* and possibly *Ae. Albopictus* (2–4). The number of DHF cases reported by the member states in three WHO regions increased from 2.2 million in 2010 to 3.2 million in 2015 (5, 6). Four viral serotypes of the Flavivirus genus cause DHF in proportions that change unpredictably over time, location, and regions country-wide (7). In Thailand, DHF cases first occurred in Bangkok in 1979 and DHF epidemics of increasing magnitude and severity occurred every two to four years beyond the endemic levels (8). Southern Thailand has the highest DHF incidence and fatality rate in Thailand (DHF incidence rate at 107.24 with fatality rate at 0.12 cases per 100,000 population) (9).

The DHF vaccine clinical landscape is very dynamic. Despite multiple obstacles for vaccine development, tremendous progress has been made, and there is now a licensed dengue vaccine. According to WHO, one dengue vaccine has been licensed, Dengvaxia® (CYD-TDV), developed by Sanofi Pasteur (10). There is still a significant research agenda to understand the mode of action of licensed and candidate dengue vaccines, and to ensure that the vaccines do not increase the risk of DHF at any time period following vaccination (11). Consequently, it is prudent that health planners design and implement intervention programs for communities with high at-risk DHF transmission.

Geographic Information Systems (GIS) technology and spatial analysis techniques are powerful tools to describe epidemiological patterns and predict the DHF transmission, as demonstrated in various locations, including China (12), Thailand (13, 14), Mexico (6), Spain (15) and Colombia (16). Determining the underlying patterns of space-time clustering (17, 18) provides an opportunity to identify DHF transmission and gain a better understanding of the disease spread (6, 18). The Google Maps™ technology has revolutionized the traditional way of using geospatial information system. Now, the technology is user-friendly for the general public, which previously was extremely detailed for highly-skilled domain experts (19, 20), therefore enabling a great potential for improving public health with a freely accessible tool (21). The web-based system can be accessed from anywhere via the internet with significantly lower deployment costs. Web browsers are available for most popular hardware and operating systems and provide access to a variety of servers. It is not necessary to install vendor-specific database access drivers on individual client computers, thereby lowering both licensing costs and administrative overhead (13).

In this study, the researchers aimed to develop an Online Advanced Analytical Service: Profiles for Dengue Hemorrhagic Fever Transmission (OSD) in Southern Thailand, and to provide the tools for uploading data, data analysis, and data display on Google Maps™.

## Materials and Methods

### System Architecture

**Fig. 1:**
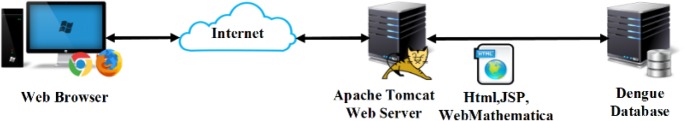
System architecture of OSD

The system was developed using JavaServer Pages (JSP) technology and web*Mathematica* technology, while Apache Tomcat and Microsoft SQL Server were used as the web server and database management system respectively (Fig. 1).

JSP technology allows developers to easily create web content that has both static and dynamic components. Additionally, JSP technology makes available complete dynamic capabilities of Java Servlet technology; as a result, it provides a more natural approach to creating static content. The main features of JSP technology are 1) a language for developing JSP pages, which are text-based documents that describe how to process a request and construct a response, 2) an expression language for accessing server-side objects and 3) mechanisms for defining extensions to the JSP language. JSP technology also contains an API that is used by developers of web containers (22). The web*Mathematica* adds interactive calculations and visualizations to websites by integrating *Mathematica* with the latest web server technology. Also, web*Mathematica* and *Mathematica* use the same underlying engine, however, they provide fundamentally different user interfaces and are aimed at different types of users. Next, web*Mathematica* offers access to specific *Mathematica* applications through a web browser or other web clients. The standard interface provided requires little training to use it effectively. Consequently, users do not require familiarity with *Mathematica* processing. Similarly, web*Mathematica* developers need only a basic knowledge of HTML and *Mathematica* to create complete, full-featured websites. In contrast, other technical programs require Java programming skills and only allow creation of small applets. Additionally, web*Mathematica* can access the full range of *Mathematica*'s built-in computational abilities; thus, developers do not work with extra code libraries (23).

In OSD, JSP script is used to work with web-*Mathematica* for contacting the Apache Tomcat and Microsoft SQL Server. The data will be retrieved from the Microsoft SQL Server database by JSP scripts in dynamic webpages with graphic images and advanced statistical tools. Accordingly, JSP scripts are used to work with Google API technology for GIS tools with data display by overlays on Google Maps™.

### Data Collection

**Fig. 2:**
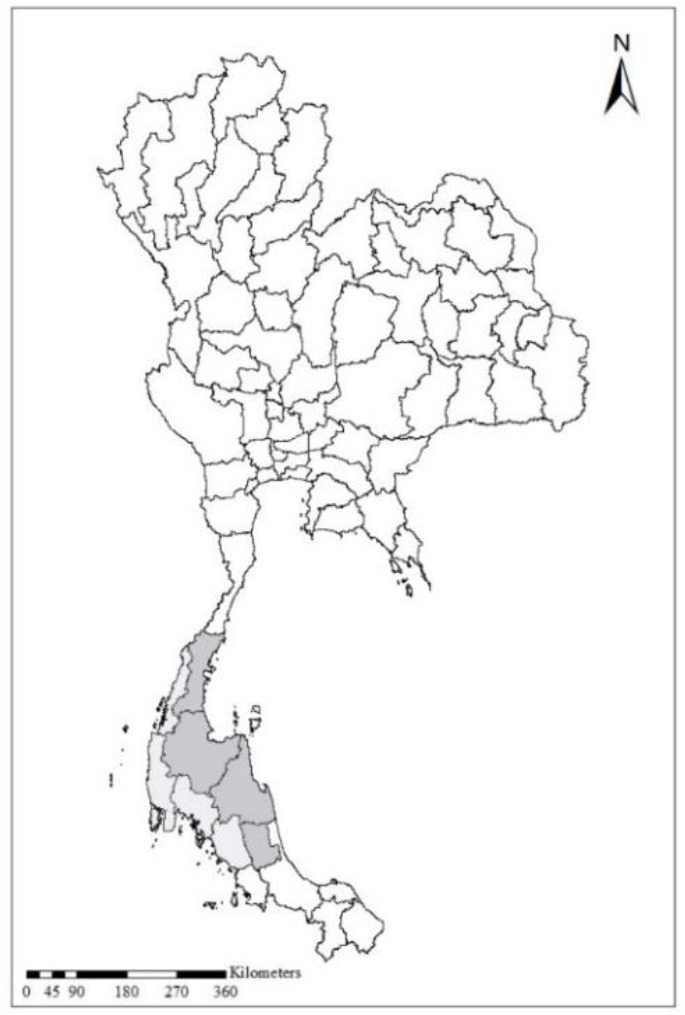
Map of Southern Thailand: Gulf of Thailand (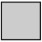), Andaman Coast (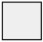)

The researchers obtained the computerized data set on monthly DHF cases in Southern Thailand by province (Gulf of Thailand: Chumphon, Surat Thani, Nakhon Si Thammarat, Phatthalung; Andaman Sea: Krabi, Trang, Phang-Nga, Phuket, Ranong) (Fig. 2) from 1981 to 2013 prepared by the Bureau of Epidemiology, Department of Disease Control (DDC), Ministry of Public Health (MOPH) (24). The geographic coordinates (i.e. latitude and longitude) of provinces were processed by Google Maps™ API.

### Evaluation of user satisfaction

The OSD system was designed for the office public health staff in Thailand. There are 11 questions for testing the OSD’s user satisfaction. Each statement was scored from 1–5 using a five point Likert scale. The researchers randomly selected 60 staff officers from public health office in Southern Thailand.

## Results

### System description

**Fig. 3:**
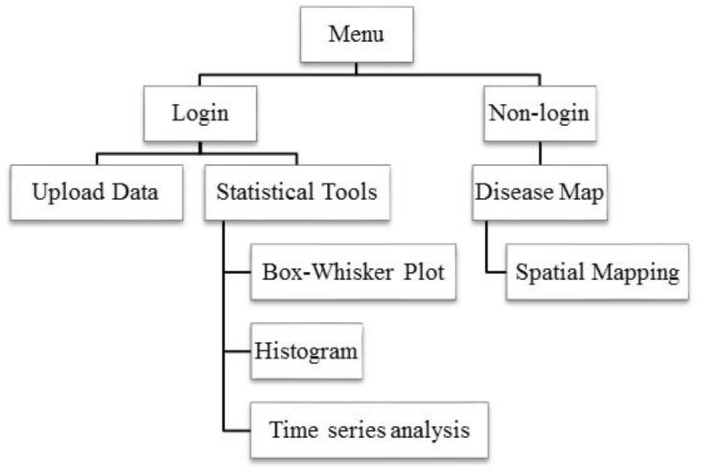
Summary functions of OSD

**Fig. 4:**
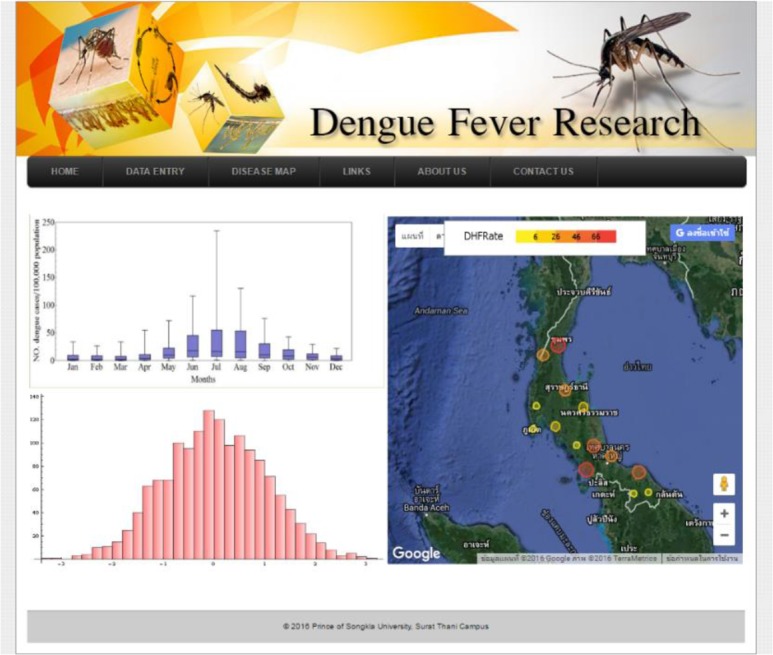
OSD home page

The OSD was designed for two groups of users: researchers and general users. The system allows access with or without login. For security reasons, the login system is available only with a username and password for a social network (i.e., Facebook account). Following the login, users can choose for two main functions: data entry and data analysis. Users not logged in have access to general information (Fig. 3). Since 2015, OSD has been available online at URL http://www.scm.co/dengue (Fig. 4).

### Data entry suite

**Fig. 5:**
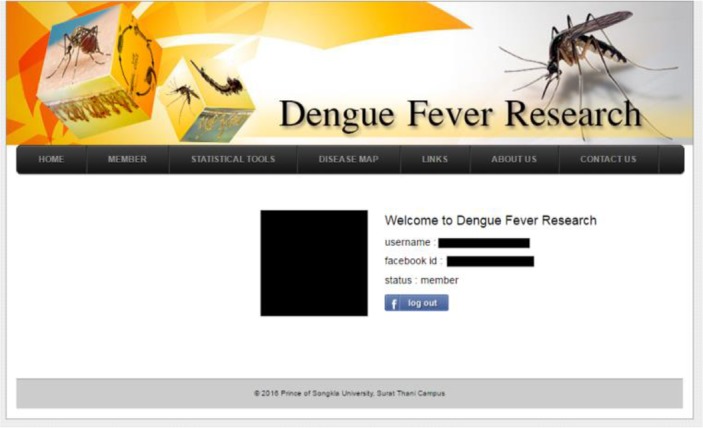
User login page via Facebook account

The data entry suite is provided to users logged in via Facebook (Fig. 5). After the login, the system allows the user to access the user information and the logout button appears in the interface (Fig. 5). Moreover, the system provides pages for uploading data to the OSD server in the CSV file format. CSV (comma separated values) is a simple file format that allows data to be saved as a table from a spreadsheet or a database. Files in CSV format can typically imported to, and or exported from programs that store data in tables, such as Microsoft Excel or OpenOffice Calc.

### Data analysis tools

**Fig. 6:**
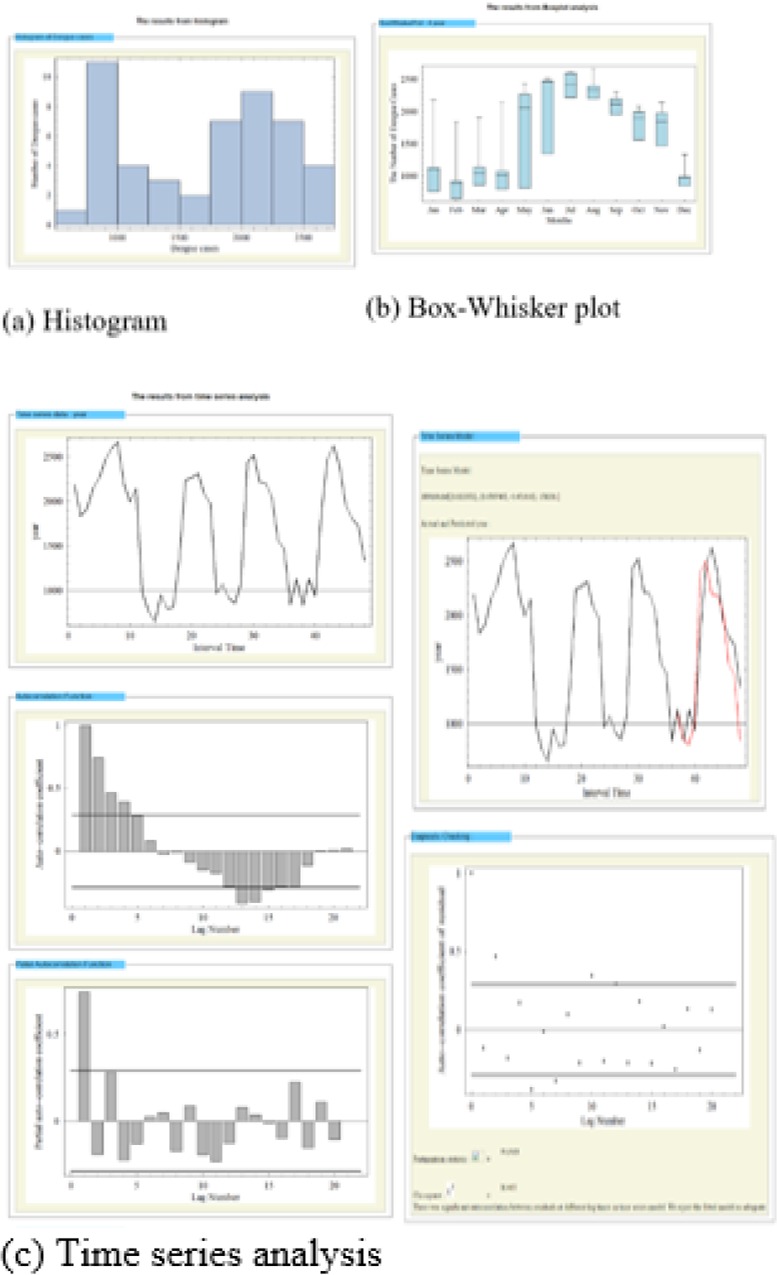
Graphical results from data analysis tool: (a) histogram, (b) box-whisker plot, and (c) time series analysis

The data analysis tools are provided on pages for real-time online data analysis with box-whisker plot, histogram and time series analysis. Users can examine distributions of DHF cases in an area using histogram (Fig. 6a) and compare the numbers of DHF cases at different times using boxplot (Fig. 6b). Additionally, these tools can create a predictive model and validate the model using univariate time series analysis method including auto-correlation coefficient and partial auto-correlation coefficient (Fig. 6c).

### Spatial Data Visualization tools: Disease Map

**Fig. 7:**
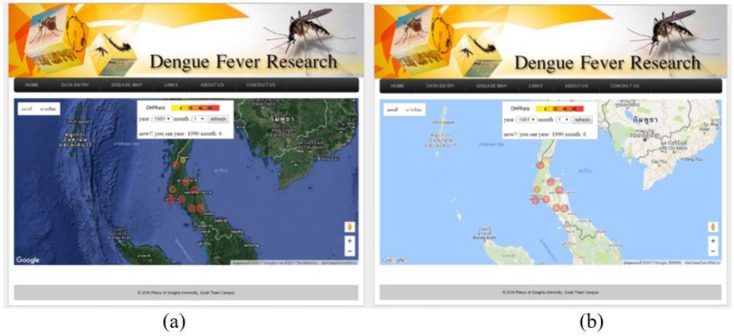
Data Display on Google Maps™ with (a) satellite imagery and (b) street map view

The spatial display tools are provided for showing the DHF distribution with transparent color circles overlaid on Google Maps™. The map displays the spatial information of DHF cases by province in the Gulf of Thailand and Andaman Coast for the yearly period of 1981 to 2013. Thus, the users can compare the spatial distributions of DHF cases in these areas by selecting the year and month in the input form (Fig. 7).

### Evaluation of OSD’s user satisfaction

The result in Table 1 shows the quantitative study derived from the evaluation of OSD’s user satisfaction of the 60 staff officers. The mean and standard deviation results for each statement, in a five point Likert scale are also shown in Table 1. The staff appeared satisfied with the quality of online tools in OSD including the materials in the system, online tools and application for the DHF research and related fields (Table 1).

**Table 1:** Evaluation of OSD’s user satisfaction. Each statement was given a score of 1–5 using a five point Likert scale

***Evaluation of OSD’s user satisfaction***	***Mean ± SD***
The quality of online tools (text and image)	4.350 ± 0.577
The consistency of online tools and engaging research on DHF	4.617 ± 0.490
The meaning of information is easy to understand	4.200 ± 0.403
The application for engaging research in related fields	4.350 ± 0.481
The knowledge on specific vocabulary on dengue research	4.317 ± 0.567
The usefulness of online data entry suite	4.183 ± 0.651
The usefulness of online data analysis tool	4.333 ± 0.475
The usefulness of online display on dengue cases overlay on Google Maps™	4.517 ± 0.504
OSD can gain insight for discovery of new knowledge on DHF	4.600 ± 0.494
OSD can help you need to study on DHF	4.733 ± 0.446
The usefulness of OSD for engaging research on DHF and related field	4.800 ± 0.403

## Discussion

A major focus of this study examined the usefulness of OSD in format display for the spatial distribution of DHF cases on maps of Southern Thailand, specifically in the provinces located by the Gulf of Thailand or the Andaman Coast. The insights gained with spatial analytical methods can generate disease distribution maps (25). Accordingly, web-based and other information technologies, including GIS, are increasingly important for studies of DHF in countries such as Thailand (13, 14), the United States of America (25), and China (26). Additionally, GIS technology integrated with mathematical and statistical models (including spatio-temporal modelling) are widely used to predict the incidences of DHF in countries such as China (12), Thailand (13, 14), Mexico (6), Spain (15) and Colombia (16). The researchers previous studies on time-series modelling have been used to predict the incidence of DHF in specific locations within Thailand (13, 14, 27).

With innovative advances and applications in technology, in 1992, Thailand’s Ministry of Public Health and the Ministry of Education integrated information regarding DHF into the national primary school curriculum (28). Since 2007 the Mosquito Online Advanced Analytic Service (MOAAS) has been used by schools in Thailand to study mosquitos (13). The researchers’ previous work (13) presented the MOAAS integrated with Google Earth™ and Google Maps™ to display geospatial information, showing mosquito larval distribution in 3D bar charts using interactive maps in Google Earth™ and Google Maps™; thus enabling scientists, teachers, and students to anticipate DHF high-risk areas. In this study, the Google Maps™ included spatial information on DHF cases in Southern Thailand by province. Therefore, the spatial distributions of DHF cases can be compared between Andaman Coast and Gulf of Thailand using the Google Maps™. Additionally, with the spatial data display tools: Disease Map, monthly case of DHF by province can artfully display as a transparent color circles overlay on Google Maps™.

Another major focus of this research was to provide identification of high-risk areas that would enable local health departments to formulate their public health strategies, initiate early preventive measures, and conduct enhanced surveillance, thereby reducing the risk of epidemics (29, 30). The system was evaluated by the users by randomly selected staff officers from public health offices in Southern Thailand. The staff officers reported satisfaction with the system’s effectiveness for relevant information in displaying the distribution of DHF cases in the community.

## Conclusion

An Online Advanced Analytical Service: Profiles for Dengue Hemorrhagic Fever Transmission (OSD) system enables identification of relevantly and graphically, spatial high-risk areas of DHF transmission in Southern Thailand. Consequently, OSD empowers public health officials to assess the effectiveness of two interventions 1) to formulate the most effective plan for disease control and include comprehensive coverage of the entire area at risk; or 2) to focusing attention on suggested or known spatio-temporal distributional trends.

## Ethical considerations

Ethical issues (Including plagiarism, informed consent, misconduct, data fabrication and/or falsification, double publication and/or submission, redundancy, etc.) have been completely observed by the authors.
